# Inspiration by estimation – how Terry Erwin affected my entomophilic attitude and most likely that of many other amateur entomologists

**DOI:** 10.3897/zookeys.1044.62666

**Published:** 2021-06-16

**Authors:** Joachim Burkhard Grammer

**Affiliations:** 1 Marsstraße 13, 85221, Dachau, Germany Unaffiliated Dachau Germany

## Abstract

Terry Erwin's influence on amateur entomologists is described by means of a personal experience form a field trip to Ecuador in 1988.

It was in the winter semester 1987/1988 when I found a one-page notice on the bulletin board in the Institute for Zoology of the University of Tübingen, Germany, announcing a “Seminar of Tropical Ecology”. The seminar would culminate with a field trip to Ecuador including a maximum number of 6–8 participants, i.e., a small group of “chosen ones”. In the fourth year of my biology degree, I was instantly enthusiastic about the idea of traveling to Ecuador, although I had no idea how I would be able to afford it. Also, the competition to go would be intense. On the first day of the seminar, it was clear that all students in line at the door to the seminar room wanted to visit the country named after the equator running through it.

After the plans for the seminar and the “general conditions” for possible participation in the trip to South America had been discussed, the work of the seminar began. On the agenda were a review of literature about tropical ecology and threats to and conservation of fauna and flora, which were of particular interest to me. A central goal of the excursion to Ecuador would be to build a platform on a tall tree on which we could stay overnight and explore a rainforest canopy. Ensuring the safety of climbing techniques using ropes, seat belts, and clamps (Perry 1978; Perry and Williams 1981) suggested that an experienced mountaineer should also be part of the team.

My literature search quickly guided me to Terry Erwin’s publications (e.g., Erwin 1983a, b), including his “Fogging Studies” in Panama (Erwin and Scott 1980). I was fascinated to learn that Erwin had subjected his field data to various calculations and extrapolations to estimate that there were 30 million different species of arthropods in neotropical rainforests, although only approximately 1.5 million insect species had been described at the time (Erwin 1982). His studies quickly became a topic for enthusiastic discussion in the seminar. For example, seminar documents that I had stored in the attic at home included a drawing of Erwin’s fogging and collecting technique (Fig. 1). The manifold reactions that Erwin’s estimates triggered among scientists will not be discussed further here. Nonetheless, these estimates and the prospect of eventually traveling to Ecuador and encountering tropical biodiversity for the first time with my own eyes had captivated me, and would not let me go.

**Figure 1.**
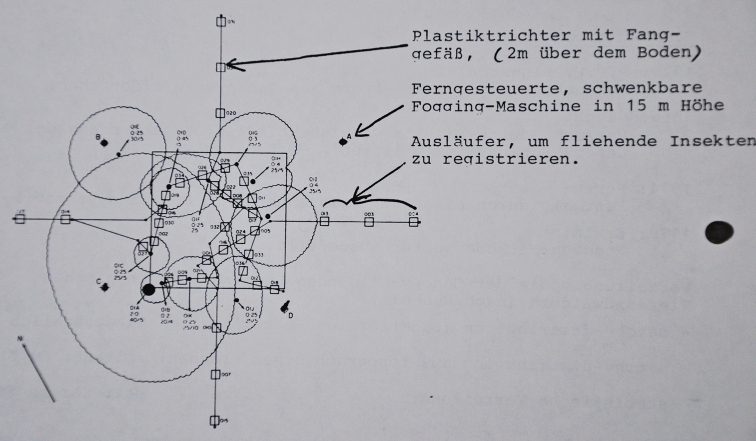
Schematic drawing of Erwin’s biodegradable insecticide fogging technology from the seminar. The letters A, B, C, and D in the sketch mark rotatable fogging machines. The small squares label plastic funnels with attached vessels that are placed ca. two meters above the ground to collect falling specimens.

The plane ride of our team, the excursion leader, a mountaineer, and six other students from Frankfurt to Quito in February 1988 marked the beginning of several weeks of adventure. Those memories have remained unforgettable to this day.

Our seminar team spent one week on the west side of the Andes and four weeks in the “Oriente” of Ecuador as guests of a Quechua family in the small village of Santa Rosa de Otas, approximately 2.5 hours downstream from the small city of Misahualli. From this location on the south bank of the Napo River, we explored the typical lowland rain forest of that region. After several days we became used to the overwhelming and omnipresent green. As our eyes became more trained to our surroundings, a variety of life revealed itself, fascinating and amazing us again and again. Terry Erwin’s estimate of the total number of insect species suddenly seemed not as exaggerated as it had seemed to some. As if to underscore the plausibility of this number, no insect or spider we spotted was like the next one we would see a few moments later (Fig. 2). Although all of us pursued our own seminar project, the “danger” of distraction was everywhere.

**Figure 2.**
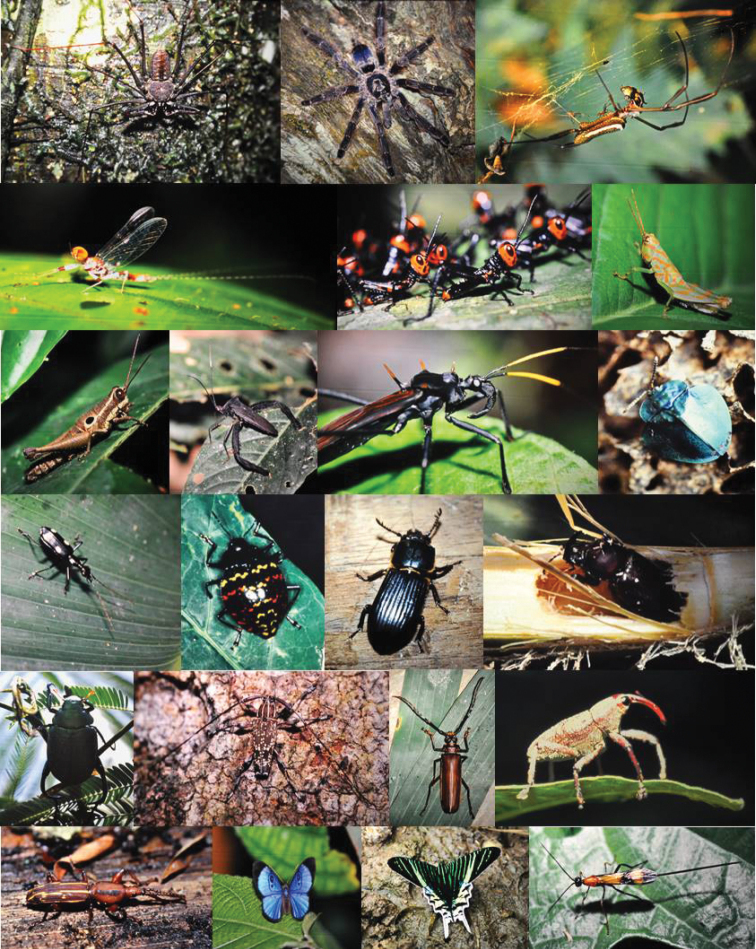
We observed a staggering variety of insect life. This is a kaleidoscopic view of arthropod diversity at Santa Rosa de Otas, Ecuador, Napo province (All photographs by J.B. Grammer during the seminar and are here reproduced from slides).

Assisted by our hosts, we assembled a platform at a height of approximately 20 meters in a large tree (Fig. 3A, B). With a little practice in the climbing technique (Fig. 3B), all participants were able to use the platform. From there, we recorded animal sounds with directional microphones, set up flight interception traps for insects at various heights, and collected samples of arthropods using nets. These included a variety of orthopterans, hemipterans (including triatomine bugs, some species of which can transmit American trypanosomiasis, also known as Chagas disease), and coleopterans. We excavated rotten logs searching for insects and collected specimens from vegetation. Because the species-level taxonomy was beyond us and likely impossible, given lack of required taxonomic literature and presence of undescribed species, we documented most of the forest life photographically (e.g., Fig. 2). And over and over again I thought of “Erwin’s number”.

**Figure 3.**
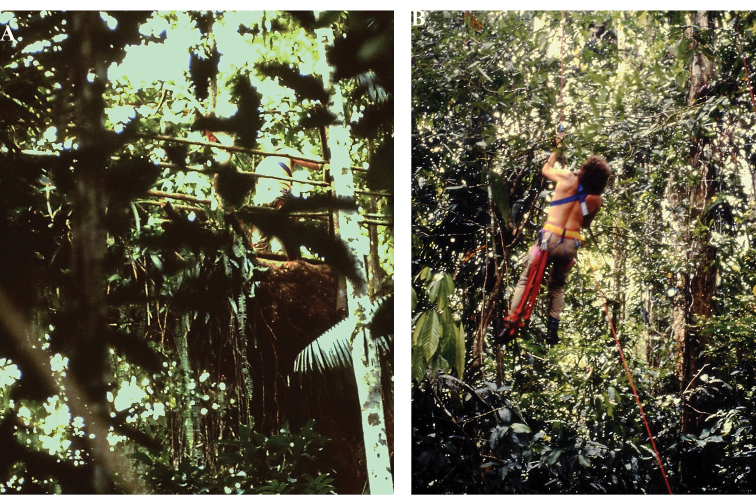
In the trees **A** our platform ca. 20 meters above ground in a tree near Santa Rosa de Otas **B** practicing the climbing technique (photographs by B. Krauss).

After returning from Ecuador we organized and evaluated our information. In the summer of 1988, we prepared a public exhibition at the Institute for Zoology of the University of Tübingen, Germany to present our results. We reported on our Ecuadorian “moments of fascination” in regional magazines (Fig. 4) and the local press.

**Figure 4.**
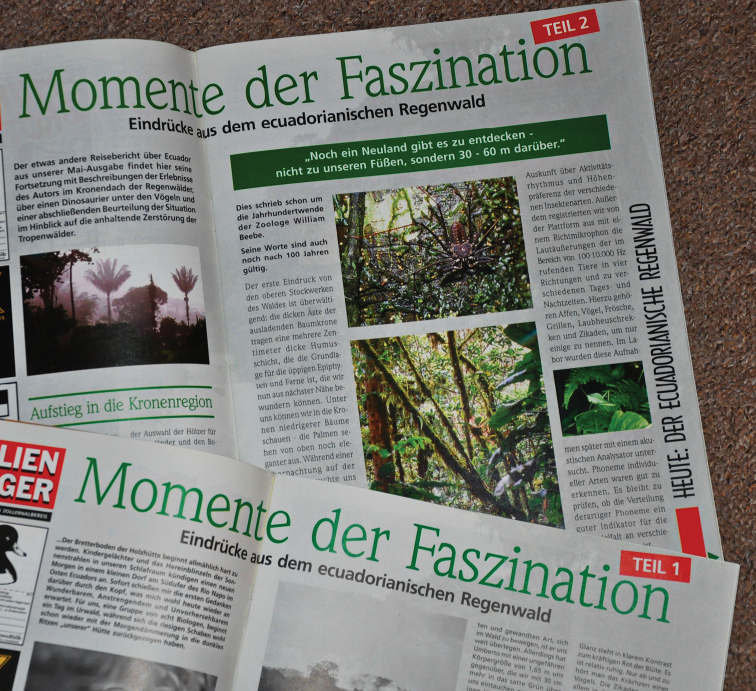
Reports of our “Moments of Fascination” in a regional magazine.

More than 30 years have passed since that seminar experience. Although my subsequent professional path led me to biomedicine, Terry Erwin’s work and the experiences in Ecuador were both the starting point and source of motivation for me to continue working as an amateur entomologist to this day. As a result, I work on my collection (Fig. 5) and am occasionally involved in publications of entomological observations. For example, this year some photographs and data about dynastine scarab beetles we had collected during our field trip to Ecuador, as well as my compilation about Ecuadorian dynastine scarabs in the Bavarian State Collection for Zoology in Munich, Germany, found their way into “The Dynastine Scarab Beetles of Ecuador” (Ratcliffe et al. 2020).

**Figure 5.**
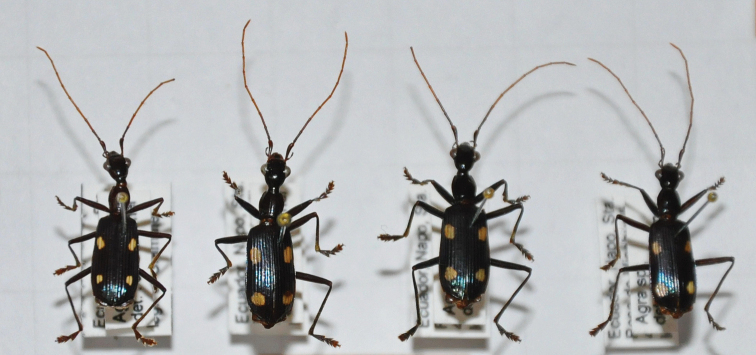
Undetermined *Agra* (Erwin’s favorite carabids) specimens from Ecuador in my collection.

I did not have the good fortune and honor to meet Terry Erwin personally, but I am grateful for all I have learnt from his work and everything I have read about him and his personality (Pensoft Editorial Office 2015; Rice 2015; D‘Souza 2020; García-Robledo et al. 2020; Kavanaugh 2020). Erwin’s work continuously inspires my interest in the world of insects. I do not think I am leaning far out of the window in saying that many professional and amateur entomologists feel the same way. In the German language, the term “amateur” has something of a condescending flavor, suggesting something unprofessional. Nonetheless the word ‘amateur’ flows from the Latin word amatio, meaning love, flirtatiousness, or dalliance. In French, an ‘amateur’ (= lover) is one who does something with love and passion. Without any doubt, Erwin expressed a deep love of insects in his work and passed on this love to his entomological colleagues and to many amateur entomologists like me. Thank you, Terry Erwin, for this gift!
